# Predictors of Biochemical Recurrence in Patients with Positive Surgical Margin After Radical Prostatectomy

**DOI:** 10.5152/tud.2025.25052

**Published:** 2025-12-05

**Authors:** Osama Mahmuod, Mulham Al-Nader, Andreas Grevendieck, Ulrich Krafft, Christopher Darr, Lukas Puellen, Jan Philipp Radtke, Andrej Panic

**Affiliations:** 1 Department of Urology, University Hospital Essen, Essen, Germany; 2 Department of Urology, Qena Faculty of Medicine, South Valley University, Qena, Egypt; 3 Department of Urology, University Hospital Düsseldorf, Germany; 4 Uroviva - Specialized Clinic for Urology, Bülach, Switzerland

**Keywords:** Biochemical recurrence, prostate cancer, prostate size, radical prostatectomy

## Abstract

**Objective:**

To investigate the predictors of biochemical recurrence (BCR) in patients with positive surgical margins (PSMs) after radical prostatectomy (RP).

**Methods:**

The data of patients who underwent open RP between 2003 and 2011 were reviewed. Only patients with PSM and negative lymph node invasion were considered for further analysis. Multivariable Cox regression analysis was performed to evaluate the correlation between clinicopathologic criteria and BCR.

**Results:**

Out of 116 patients, 80 (69%) developed BCR with a median (interquartile range [IQR]) time to recurrence of 19 (9-50) months. Median (IQR) time of follow-up in non-recurrent patients was 121 (47-148) months. The 5- and 10-year BCR-free survival rates were 43% and 28%, respectively. Complete data regarding margin parameters were available only for 98 patients, of which 71 (72%) developed BCR. Univariable analysis demonstrated that prostate volume (PV) (HR 0.97, 95% CI 0.95-0.99, *P* = .005), highest Gleason grade (GG) at the margin (HR 1.73, 95% CI: 1-2.83, *P* = .028 for GG 4-5 vs. 3), tumor GG group 2 (HR [Hazard Ratio] 2, 95% CI 1.09-3.94, *P* = .025) and 4-5 (HR 2.34, 95% CI 1-4.98, *P *= .028) were significant predictors of BCR. In multivariable analysis, only PV remained an independent predictor of BCR (HR 0.98, 95% CI 0.96-0.99, *P* = .03).

**Conclusion:**

In patients with PSM after RP, smaller prostates have a higher probability of BCR. Further studies are needed to investigate this association. The highest GG at the margin has important predictive accuracy and should be reported in the pathology report.

**Main Points**

- The identification of men at high risk of early recurrence within the subgroup with a positive surgical margin after radical prostatectomy is crucial for targeted adjuvant therapy.
- Margin characteristics, particularly the highest Gleason grade at the margin, are significant predictors for biochemical recurrence (BCR).
- Patients with smaller prostates have a significantly higher risk of BCR than those with larger prostates.

## Introduction

Although a positive surgical margin (PSM) after radical prostatectomy (RP) is considered an unfavorable pathologic feature with worse oncologic outcomes, in some large studies up to 45% of patients with PSM do not develop tumor recurrence at long-term follow-up.^1-3^ Management of PSM remains a dilemma as a clear consensus is still lacking. In the ARTISTIC (a prospective meta-analysis of RADICALS, RAVES, and GETUG-17) meta-analysis including the results of 3 randomized trials (RADICALS-RT (a phase III trial of adjuvant radiotherapy vs observation+salvage radiotherapy after radical prostatectomy), GETUG-17(a randomised, phase III trial of adjuvant radiotherapy versus early salvage radiotherapy plus short-term androgen deprivation therapy in men with localised prostate cancer after radical prostatectomy), and Adjuvant Versus Early Salvage [RAVES]), adjuvant radiotherapy (RT) did not improve the biochemical recurrence (BCR)-free survival (HR = 0.95, 95% CI = 0.75-1.21, *P* = .70) compared to salvage RT. Most patients were in the high-risk category, including 71% with PSM, suggesting that marginal status alone is not sufficient to recommend adjuvant RT.^4^ The decision may be easier by the presence of other associated unfavorable pathologic features, such as pT3b disease and Gleason-group 4-5, for which adjuvant therapy is recommended in many reports.^5^ On the other hand, the management is challenging in low-risk PSM cohorts, i.e., patients with lower Gleason score (GS) or organ-confined disease. This gray area may also extend to some subsets of pT3a disease, which in some studies had a comparable prognosis to organ-confined disease.^6^ In a recent study of more than 20 000 patients, pT2 and pT3a diseases were seen to have almost the same 20 years BCR-free survival rates (57% vs. 57%, respectively) in patients with PSM and Gleason grade group (GGG) 1-2; whereas in the presence of T3b disease, BCR-free survival drops significantly to 38%.^6^

Identifying men at higher risk for early recurrence in a PSM subgroup is imperative both to select men who might benefit most from adjuvant treatment and to avoid overtreatment and radiation toxicity in lower-risk cohorts. In the last decade, many large RP series have shown an association between surgical marginal parameters such as location, number, length, Gleason pattern, and BCR.^2,7-9^ However, their use in clinical practice remains insufficient due to the lack of well-established risk models that consider all available risk factors. Due to this lack in the literature, a retrospective analysis was performed to investigate the prognostic factors of BCR in the subgroup of RP.

## Materials and Methods

### Patient Cohort

This retrospective analysis was conducted in accordance with the Declaration of Helsinki (as revised in 2013) and was approved by the institutional ethics board of the University of Duisburg-Essen (approval no.: 15-6704-BO). Informed consent was waived by the institutional review board in view of the retrospective nature of the study. All the procedures being performed were part of the routine care.

The data of patients who underwent retropubic RP with or without pelvic lymph node dissection between 2003 and 2011 were reviewed. Unless there was an intraoperative contraindication, nerve-sparing RP with preservation of the tip of the seminal vesicle was the standard of care at the institution at that time. All surgeries were performed by a single experienced surgeon with more than 20 years of experience and over 1000 performed RP procedures.

Only patients with PSM were considered for further analysis. All patients who received hormonal treatment or RT before surgery were excluded from analysis. Prospectively documented clinical data were recorded, including age, preoperative prostate specific antigen (PSA), and prostate volume (PV) calculated by transrectal ultrasound.

### Histopathological Analysis

After histopathological examination by the same team of experienced uropathologists, the pathological data were retrospectively recorded, including stage (TNM [Tumor Node Metastasis]), GGG, tumor volume, perineural invasion, and nodal status, and the criteria of surgical margins including location, number, length, and highest Gleason pattern of PSM were also documented. A PSM was defined as tumor cells approaching the inked margin.^10^ The highest GGG of the tumor at PSM was assessed within the tumor focus contacting the inked margin; PSM length was defined as the total length of the tumor in contact with the inked margin. The added length of all margins was recorded by the presence of multiple positive margins.

### Follow-Up and Definition of Biochemical Recurrence

The follow-up data were also collected to identify those men who developed BCR. Only patients who completed a minimum of 6 months follow-up were included. Biochemical recurrence was defined as 2 consecutive PSA values >0.2 ng/mL after RP. The primary outcome of the analysis was to assess potential risk factors for BCR in patients with PSM.

### Statistical Analysis

For the descriptive statistics, median and interquartile range (IQR) or mean with (SD) are used to represent continuous variables, while frequencies and proportions are used to represent categorical variables. A comparison of mean values was performed using the Student *t*-test and a comparison of medians was performed using the Mann–Whitney *U*-test. Chi-square test was used for comparing categorical variables. Biochemical recurrence–free survival was defined as the interval between surgery and date of recurrence, and those without BCR were censored at the last follow-up visit. In patients with complete data on marginal characteristics (localization, length, and GS), univariable Cox regression analysis was performed to evaluate the correlation between clinicopathologic criteria and BCR. In addition, confounding factors identified in the univariable analysis were further examined in a multivariable Cox proportional hazard model. Patients’ clinical data, including PSA, age, PV, and pathological tumor volume, were used as continuous variables in the multivariate analysis, whereas the other pathological data were modeled as categorical variables. *P*-values ≤ .05 indicate significance. All statistical analyses were performed using SPSS version 16 software (SPSS Inc.; Chicago, IL, USA).

## Results

We identified 135 (20%) with PSM out of a total of 689 patients. Follow-up data were available for 123 patients. Seven patients who had lymph node metastases or had received adjuvant RT or hormonal therapy wer excluded. Overall, a cohort of 116 patients was available for final analysis. The median (IQR) preoperative PSA was 8.3 (5.6-13.7) ng/mL and the mean (SD) PV was 38.5 (13.8) mL. In the RP specimen, the GGG 1, 2, 3, 4-5 were 25%, 41.3%, 18.1%, and 15.6%, respectively. The pathologic tumor stage was pT2 in 61.2% and pT3 in 38.8% (Table 1). Eighty (69%) patients developed BCR with a median (IQR) time to recurrence of 19 (9-50) months. Median (IQR) time of follow-up in non-recurrent patients was 121 (47-148) months. The 5- and 10-year BCR-free survival rates were 43% and 28%, respectively.

Complete data regarding margin parameters (site, length, and highest Gleason grade [GG]) were available only for 98 patients. The median length of the PSM was 3.5 mm (0.5-14), and an extensive PSM of more than 3 mm in length was found in 55%. Positive surgical margin was multifocal in 31% of patients and unifocal in 69%, with the apex and posterior location being most affected in the unifocal group (48.5% and 44%, respectively). The highest Gleason pattern at the margin was mostly 3, which was recorded in 69%.

Of the 98 patients, 71 (72%) developed BCR. Univariate analysis demonstrated that PV (HR 0.97, 95% CI 0.95-0.99, *P* = .005), highest GG at the margin (HR 1.73, 95% CI 1-2.83, *P* = .028 for Gleason 4-5 vs. 3), tumor GGG 2 (HR 2.95, 95% CI 1.09-3.94, *P* = .025) and 4-5 (HR 2.34, 95% CI 1-4.98, *P* = .028) were significant predictors of BCR. By adjusting the aforementioned predictors in multivariate analysis, only PV remained an independent predictor of BCR (HR 0.98, 95% CI 0.96-0.99, *P* = .03) (Table 2).

**Figure 1.**
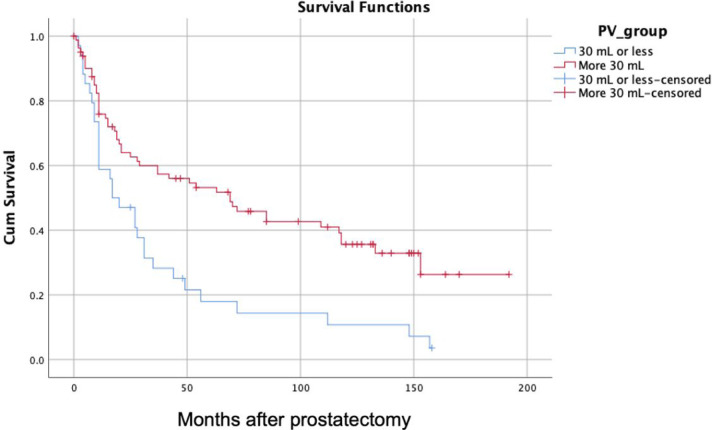
Biochemical recurrence–free survival curve stratified by the prostate volume (≤30 and >30).

Among the total cohort of 116 patients, using Receiver Operating Characteristic (ROC) curve analysis and Youden’s Index, the cutoff PV for predicting BCR was 30 mL. By stratifying patients based on this cutoff size, Kaplan–Meier curves demonstrated significantly lower BCR-free survival in the group of patients with small PV (*P* = .001). The 5- and 10-year rates of BCR-free survival were 18 and 11%, respectively, for PV ≤ 30 (34 patients) versus 53 and 36%, respectively, for patients with PV > 30 mL (79 patients) (Figure 1).

## Discussion

Rates of PSM published in the literature vary between 10% and 40% depending on tumor stage, grade, surgeon, and surgical technique.^10,11^ These results are consistent with the literature; a PSM rate of 17% was found in 689 patients. Identifying high-risk PSM patients is of great importance to individualize follow-up protocols and identify those who may benefit from adjuvant RT. In analyzing this subset of PSM, a strong association was demonstrated between PV and recurrence risk. When PV was adjusted as a continuous parameter for other confounding factors related to either overall clinicopathologic parameters or marginal features, increasing PV was associated with a lower likelihood of BCR (HR 0.98, 95% CI 0.96-0.99, *P*= .03). Similarly, but in a whole RP cohort, an early study reported poor BCR-free 4-year survival of 78% in PV < 30 versus 88% in PV 30 to 75 mL (*P* = .012) in men with PSA less than 10 ng/mL; moreover, BCR-free survival in a PSA group between 10 and 20 ng/mL decreased dramatically to 25% versus 60% in the same PV groups (*P *= .012), respectively. Interestingly, no patients with PV more than 75 mL developed a BCR.^12^ In another study, at a median follow-up of 20-25 months, patients with large prostates of >75 g were also less likely to suffer BCR (5% vs 24%, *P *< .001).^13^ These findings were explained by lead-time bias in the aforementioned studies, as high PSA in large prostates may trigger early biopsy and diagnosis of Prostate Cancer (PCa), leading to a lower stage and rate of PSM after RP and thus a better oncologic outcome. Unfortunately, it could not be assessed in this series. Sooriakumaran et al^14^ found another interesting point that smaller glands have a higher percentage of cancer, which may favor early infiltration of the capsule and higher incidence of PSM after surgery.

The previous theories could explain the higher stage in small prostates but not the higher grade, which was also observed in men with small glands. Freedland et al^15^ reported a 7.5-fold higher likelihood of high-grade disease in small prostate weights (<20 g vs. 100 g), suggesting increased intrinsic tumor aggressiveness in small glands and not just late disease diagnosis. The authors of this study tried to avoid the lead-time bias; they excluded patients diagnosed only because of an elevated PSA and included those with cT2 and cT3 in the analysis. They also found that small prostate weight was an independent predictor of BCR in the multivariate analysis (RR = 11.75; 95% CI, 2.76 to 49.96; *P *< .001).

Contrary to the aforementioned studies, some large series did not observe any correlation between PV and oncological outcome. Westhofen et al^16^ matched 184 patients with PV ≥ 100 cm^3^ and 745 patients with PV < 100 cm^3^ for age, body mass index, and pT stage, and found no difference in oncologic outcome between small and large prostates. However, they used a large volume of 100 cm³ as a cutoff for comparison, which might affect the results because the effect of PV was evident in very small prostates in this study and the previous series. Mandel et al^17^ found that patients with larger PV were older, had higher preoperative PSA, and were more likely to have organ-confined disease with a higher rate of GGG 1. Small PV was only a predictor of recurrence in the univariate analysis (HR 0.995; 95% CI 0.992-0.999, *P* = .019) and not in the multivariate analysis (HR 0.996; 95% CI 0.992-1.000, *P* = .070); however, the median follow-up in this study was relatively short of 36 months. The limitations in the studies to date, either for or against the effect of PV, prevents from drawing a solid conclusion, and large prospective studies on this topic are still needed.

Many studies have attempted to identify margin characteristics that may influence the risk of subsequent BCR. Due to the retrospective nature of these studies, the heterogeneity of the parameters included in the final analysis, and the different follow-up periods, the results were inconsistent. In this study, highest GG at the margin was associated with recurrence only in univariate analysis, but this may be related to the small sample size. Kates et al^8^ found a significant association between GG at the margin and tumor aggressiveness in 405 patients, as lower grade was associated with shorter margin length (odds ratio [OR] 0.77; 95% CI 0.60-0.94) and was also associated with lower BCR risk in multivariate Cox models (HR 0.50; OR 0.25-0.97). This finding was supported by most other studies that examined the prognostic significance of PSM characteristics.^2,9^ Moreover, Preisser et al^2^ found that a GG ≥ 4 at margin versus Gleason 3 was associated not only with early BCR but also with worse cancer-specific survival at 96 months (87.1 vs. 100, *P* < .01). Positive surgical margin length and multifocality were not associated with tumor recurrence in this study; however, extensive disease has been reported to correlate with BCR in several studies. In 2008, Ochiai et al^18^ found in the PSM cohort that there was no difference in progression in patients with a PSM of 1 mm or less compared with those with a margin of 1.1-3 mm, but a significant difference was seen between margin length ≥3 mm versus <3 mm, which was confirmed in multivariable analysis; however, the study was limited by the relatively small sample size of 117 patients. Shikanov et al^19^ later reported in a large RP cohort of 2866 patients and 402 PSM that even a short PSM (≤1 mm) can lead to an unfavorable outcome in a subset of patients with high-risk disease; a short PSM was associated with a 17% lower 3-year BCR-free survival than in patients with negative margin and pT3 and GS ≥ 7. The median follow-up time in this study was only 20 months.^19^ Although the prognostic significance of margin length has been demonstrated in the literature, the prognostic value in subgroups of the pathological stage is contradictory.^20-22^ Recently, 2 large studies with long follow-up were published by Preisser et al^2,23^ The first included 576 men with PSM and organ-confined disease; higher BCR-free survival was seen in those with margin length <3 mm vs. 3 mm after 72 months of follow-up (88.4 vs. 66.3, *P* < .001).^23^ In the second study for patients with non-organ-confined disease, significantly higher BCR-free survival was demonstrated in pT3a patients with a margin of <4 mm vs. 4 mm after 96 months of follow-up in 1007 PSM patients (45% vs. 27.8%, *P* < .01).^2^ In both studies, margin length was an independent predictor of BCR. Dason et al^24^ recently found that adding a subclassification of surgical margin including length and maximum GG to the BCR prediction nomogram generated by Memorial Sloan Kettering Cancer Center improved prediction accuracy (increasing the c-index from 0.717 to 0.753) in a cohort of patients with PSM. It is important to note that the International Society of Urologic Pathology consensus conference in 2009 recommended routine reporting of Gleason pattern and length of PSM.^25^

### Limitations

The main limitation of this study is its retrospective nature and small sample size. Furthermore, the PSA was carried out in different laboratories, but the results were mostly confirmed in the lab. In addition, the data were insufficient to evaluate the predictors of metastasis-free and cancer-specific survival. Although overall GS and higher GG at the surgical margin were correlated with recurrence only in univariate analysis, these factors are still important determinants of oncologic outcome in patients with PSM. On the other hand, the present study demonstrates for the first time in the literature the correlation between the PV and tumor progression; patients with smaller prostates have a significantly increased risk of BCR compared with patients with larger prostates. This correlation could be further investigated for inclusion in risk models to predict the outcome of PSM patients after RP.

## Figures and Tables

**Table 1.** Patient Clinicopathological Features

Variable	Value
Age (years), median (IQR)	65 (60-69)
PSA (ng/mL), median (IQR)	8.3 (5.6-13.7)
Prostate volume (mL), mean (SD)	38.5 (13.8)
PSA density, median (IQR)	0.23 (0.14-0.38)
GGG, n (%) Group 1 Group 2 Group 3 Group 4-5	29 (25) 48 (41.3) 21 (18.1) 18 (15.6)
Pathological stage, n (%) T2 T3	71 (61.2) 45 (38.8)
PSM Site, n (%) Base Apex Posterior Anterior Multiple missing	2 (1.7) 41 (35.3) 31 (26.7) 3 (2.6) 35 (30.2) 4 (3.5)
PSM length, n (%) <1 mm 1-3 mm More than 3 mm Missing	6 (5.2) 38 (32.7) 54 (46.5) 18 (15.5)
PSM highest Gleason grade, n (%) 3 4-5 Missing	68 (58.6) 30 (25.9) 18 (15.6)
Removed LN, n (%) ≥ 10 < 10	30 (25.9) 86 (74.1)
Perineural invasion, n (%)	85 (73.2)
Tumor volume (mL), Median (IQR)	10 (15-20)

GGG, Gleason grade group; IQR, interquartile range; LN, lymph nodes; PSM, positive surgical margin.

**Table 2.** Univariable and Multivariable Cox Regression Analysis

Variable	Univariate Analysis HR (95 % CI)	*P*	Multivariate Analysis HR (95 % CI)	*P*
PSA	1 (0.98-1.04)	.3		
Age	0.98 (0.95-1)	.38		
Tumor volume	1 (0.98-1)	.32		
Pathological stage cT2 cT3	Ref. 1.3 (0.82-2.1)	.25		
ISUP Group 1 Group 2 Group 3 Group 4-5	Ref. 2 (1.09-3.94) 1.89 (0.88-4) 2.34 (1-4.98)	.025 .1 .028	Ref. 1.64 (0.84--3.1) 1.1 (0.48-2.91) 1.32 (0.53-3.2)	.14 .69 .54
PNI No Yes	Ref. 1.16 (0.61-2.21)	.64		
No. removed LN <10 ≥10	Ref. 1.33 (0.79-2.24)	.27		
Site, n (%) Anterior Apex Posterior Multiple Base	Ref. 0.62 (0.18-2) 0.61 (0.18-2.1) 0.56 (0.16-1.9) 0.88 (0.14-5.3)	.45 .43 .36 .89		
Highest GS in SM 3 4-5	Ref. 1.73 (1-2.83)	.028	Ref. 1.54 (0.81-2.9)	.18
Length of safety margin <1 mm 1-3 mm >3 mm	Ref. 0.85 (0.32-2.22) 0.99 (0.39-2.5)	.74 .99		
Prostate volume	0.97 (0.95-0.99)	.005	0.98 (0.96-0.99)	.03

GS, Gleason score; HR, hazards ratio; ISUP, International Society of Urological Pathology; NI, perineural invasion; SM, Surgical Margin.

## Data Availability

The data that support the findings of this study are available on request from the corresponding author.
